# Quantify total activity by volume‐of‐interest expansion with clinical SPECT/CT systems, a phantom study

**DOI:** 10.1002/acm2.13828

**Published:** 2022-11-08

**Authors:** Junguo Bian, Judy R James, Robert Wagner, James Halama

**Affiliations:** ^1^ Department of Radiology Loyola University Medical Center Maywood Illinois USA

**Keywords:** activity estimation, quantitative SPECT/CT, recovery coefficients

## Abstract

**Purpose:**

Quantitative measurements of activity in SPECT are important for radioisotope therapy planning and disease diagnosis. The aim of this manuscript is to develop a robust method to quantify the total activity in a volume‐of‐interest (VOI) of different quantitative SPECT reconstructions and validate the estimation accuracy.

**Methods:**

We customized an IEC body phantom using 3D printing technology and made six sphere inserts of 1–6 cm in diameter with at least 3 cm separation. The activity concentration within the spheres was in the range of patient lesion/organ activity. The background activity was then increased from zero to a sphere/background activity concentration of 8:1, 4:1, and 2:1. SPECT data were acquired with Philips Brightview and GE Discovery 670 SPECT/computed tomography (CT) systems under clinical acquisition protocols. Quantitative SPECT images were reconstructed with Hermes SUV‐SPECT (both Philips and GE data) and GE Q.Metrix (GE data only). The quantitative SPECT reconstructions are iterative with scatter, CT attenuation correction, and resolution recovery. We quantified the total activity by expanding the sphere VOI to include a spill‐out region. Background correction was applied by sampling a region outside the spill‐out region. The true fractions (TFs) (total activity/true activity) were measured for all six spheres for all SPECT acquisitions.

**Results:**

The TF is close to 100% for 2–6 cm spheres for zero background, 8:1 and 4:1 sphere/background activity ratios. The TF was found to be unreliable for the 1‐cm sphere because of the limit of phantom design. TF accuracy for 2:1 sphere/background ratio was degraded due to significantly large background, inadequate scatter correction and detector count loss.

**Conclusions:**

The results demonstrated that the proposed quantification method is accurate for objects of different sizes in currently clinical quantitative reconstruction and has the potential for improving the accuracy for therapeutic treatment planning or radiation dosimetry calculations.

## 1 INTRODUCTION

Similar to computed tomography (CT), emission tomography is widely used in clinical practice and biomedical research. Emission tomography uses various radionuclide‐labeled compounds for imaging, and quantitative emission tomography can be a powerful investigation tool for both clinical and research practices. The two principal forms of emission tomography are SPECT and PET. Unlike SPECT, PET systems from the early development have been designed to produce reconstructed images that are inherently quantitative mainly because the attenuation correction for PET is relatively straightforward to apply, and in early two‐dimensional PET scanners, the amount of scattered radiation detected was low. Modern PET scanners are always paired with CT scanners for both attenuation and scatter correction, and clinically, there are almost no stand‐alone PET scanners. PET images have since been used quantitatively, and there are manufacture procedures to validate the quantitative accuracy of PET images.

In SPECT, attenuation correction and scatter correction have been more challenging. The accurate correction of attenuation and scatter also requires that transmission data (CT) is available. However, the incorporation of CT into SPECT is not as widely available as PET/CT. Nevertheless, new SPECT systems come with CT. With the combined SPECT/CT scanners and more robust iterative image reconstruction algorithms (ordered‐subset maximum‐likelihood expectation maximization algorithm combined with resolution recovery), quantitative SPECT has been under development for a long time.^1^, ^2^, ^3^ Different vendors have developed their own quantitative SPECT imaging systems for clinical use. For example, GE has a quantitative SPECT package called Q.Metrix. Hermes as a major vendor in nuclear medicine also has its own quantitative SPECT reconstruction package (standardized uptake value [SUV]‐SPECT). Both of the reconstruction packages require the availability of CT data and have incorporated scatter correction of the SPECT data.

For both PET and SPECT images, quantitative means pixel values in the concentration of the radioactivity in a volume (Bq/ml). From a quantitative PET/SPECT image, either a SUV (which is used to quantify metabolic state of tissues and tumors) or the total activity within a volume‐of‐interest (VOI) can be calculated. The SUV is a ratio of the radioactivity concentration in a VOI to the average concentration in the whole patient volume. An SUV greater than one implies active tissue incorporation of the radiotracer. Often the peak SUV in a VOI is reported. The total activity in an organ VOI is used to perform internal radiation absorbed dose calculations for a patient's whole body, organ, and/or tumors.^4^ The total activity has great implication for therapeutic treatment procedures, such as Y‐90, In‐111, Lu‐177, and I‐131 treatments for cancer, especially for quantitative SPECT.

Both PET and SPECT systems suffer from partial volume effects^5^, ^6^ as the result of the imperfect spatial resolution of these imaging systems. In general, clinical PET systems have better spatial resolution than clinical SPECT systems. We acknowledge that partial volume effects exist in PET. In this paper, we are focusing on the effects from SPECT imaging. Due to the relatively poor spatial resolution of SPECT systems, the effect is significant and leads to errors in organ activity measurements. The partial volume effect is observed as the spill‐out of activity beyond the organ/tumor boundary. If the spill‐out is not included in the organ/tumor VOI, the activity will be underestimated. To compensate for the underestimation, multiplicative recovery coefficients (a concept originated from PET) have been applied.^7^, ^8^, ^9^, ^10^, ^11^, ^12^, ^13^, ^14^, ^15^ However, as Ismail et al.^14^ pointed out, recovery coefficients are mostly measured based on spherical objects in phantoms and is size‐dependent and reconstruction‐algorithm‐dependent. There are studies to incorporate more geometry information into the calculation of recovery coefficients instead of calibration based on simple spherical objects and have achieved some degree of success.^16^ However, recovery coefficients always require some type of calibration either by experiments or simulation.

In this manuscript, we focus on the total activity estimation by expanding the VOI to include both the object region and the spill‐out region so that all of the spill‐out activities are captured. By doing so, we eliminate the need to obtain recovery coefficients to estimate the total activity of the object. The activity can then be assigned to the true volume of the object and be used for dosimetry purposes or to calculate an activity concentration for diagnostic purposes.

## 2 MATERIALS AND METHODS

### 2.1 Experiment design and data acquisition

**FIGURE 1:**
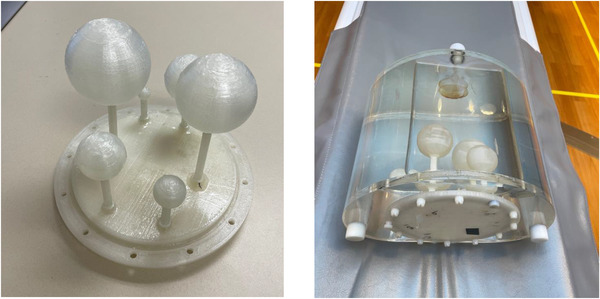
The modified IEC body phantom used in our experiment: customized IEC body phantom cover (left); phantom filled with water with the customized phantom cover (right)

Considering SPECT has relatively poor resolution compared to PET and that therapeutic application generally involves large objects such as the whole organ, we modified the IEC body phantom designed for PET system evaluation to include objects of larger size for our experiment. The IEC phantom cover is customized using 3D printing to hold six hollow spheres from 1 to 6 cm of diameters. The spheres are arranged so that all sphere surfaces are a minimum of 3 cm apart from one another. The 3 cm spacing was chosen with a consideration of the spatial resolution of SPECT systems to ensure the measurements of one sphere would not interfere with another. In order to meet this requirement, the placement of the spheres at depth inside the phantom had to be staggered because of space limit. Hollow stems are fitted for filling the spheres and inserting inside the IEC phantom. We used a 3D printer to make the cover, stems, and the spheres. The advantage of using 3D printer for the phantom is the low cost and more freedom in design. The phantom we customized is shown in Figure 1. Because of the limitation of available materials, the spheres are not transparent.

Tc‐99m activity was prepared so that the Tc‐99m concentrations in the spheres are approximately 12 μCi/ml, which is about the same concentration as specified in IEC SPECT test documents.^17^ The phantom was first scanned with no background activity under clinical acquisition protocols. Activity was then added to the background of the phantom to make the sphere/background activity concentration ratio to be 8:1, and the scan was repeated with the same clinical protocol. The activity in the background was then doubled to make the sphere/background activity concentration ratios to be 4:1 and 2:1, and the phantom was scanned with the same clinical acquisition protocols. For the sphere/background activity concentration ratios of 2:1, the background activity is actually unrealistically large for clinical practice settings but is included in the manuscript for investigational purpose.

The phantom data acquisitions were performed on a clinical GE Discovery 670 SPECT/CT and Philips Brightview SPECT/CT systems. The crystal thicknesses of the two systems are both 9 mm. For both studies, SPECT data were acquired using the LEHR collimator, a noncircular orbit under step‐and‐shoot mode. The projection view settings are 30 s per view, 128 views (for Philips) or 120 views (for GE) with a matrix size of 128 × 128, and a zoom factor 1.46. The resulted SPECT reconstruction image pixel size is about 3.2 mm with the same array dimension as the detector matrix size. The GE SPECT/CT system is incorporated with a 16‐slice diagnostic CT scanner, whereas the Philips Brightview SPECT/CT system uses a flat‐panel‐based cone‐beam CT system. The Philips Brightview does not have a quantitative reconstruction package. The Hermes SUV‐SPECT reconstruction software was used for quantitative reconstruction of Brightview data. For the GE Discovery system, both the manufacture‐provided Q.Metrix quantitative package and the Hermes SUV‐SPECT reconstruction tools were used to reconstruct quantitative SPECT images. The Q.Metrix quantification was performed on the Xeleris workstation. All the SPECT reconstructions use OSEM (16 subset for Philips or 15 subsets for GE, 5 iterations) with CT attenuation correction, scatter correction, and resolution recovery.

The quantification of the SPECT reconstruction requires the measurement of sensitivity coefficient that will be used to convert the counts into activity concentration. This coefficient is usually available from the SPECT system annual physics surveys. The details about the measurement of this coefficient have been described by Gnesin et al.,^9^ and we have followed exactly the same procedures. Image voxels from the quantitative reconstructions are expressed in units of Bq/ml.

We then measured the total activity within the spheres by placing VOI's at the boundaries of the spheres using VOI tools provided by Hermes or GE Xeleris workstation. To include the spill‐out of activity outside the boundaries of the spheres, we overlaid enlarged VOI's over each sphere. Altogether, 8 data acquisitions (two clinical systems, four acquisition for each system) were performed with the two clinical systems, and 12 sets of reconstruction images were reconstructed from the 8 data sets using Hermes and GE Q.Metrix quantitative SPECT reconstruction (GE data were reconstructed by both GE Q.Metrix and Hermes, whereas Philips data were reconstructed with Hermes only).

### 2.2 Proposed tracer quantification method

Our proposed quantification method is as follows: We first co‐registered the SPECT images with the CT image using either Hermes or GE Xeleris. We placed six VOIs that match the diameters for the hot spheres on the reconstructed SPECT images using CT images as references. This set of VOIs is called VOI1 (sphere 1). We then expanded the diameter of each sphere in VOI1 by a fixed distance (sphere 2). The volume between the two VOI's for each sphere is called VOI2. VOI2 essentially captures the spill‐out regions of Tc‐99m activities. We then expanded the diameter further by about 1 cm (sphere 3). The volume between the sphere 2 and sphere 3 is called VOI3. VOI3 will be used to estimate mean background activity concentration. Total activity is the sum of the activities within VOI1 and VOI2 minus background activity. The background activity is estimated using the mean activities within VOI3 multiplied by the volume of VOI2. The assumption here is that the background activity is only significant for the activity within VOI2. The whole quantification method can be summarized by the following equation:

$$A^{\mathrm{corr}}=A+A_{\mathrm{SP}}-V_{\mathrm{SP}}\bar{B\left(v\right)}$$

In which *A^corr^* is the final estimated total activity within the sphere, *A* is the total activity within VOI1, *A_SP_* is the total activity within VOI2, which is the spill‐out activity, *V_SP_* is the volume of VOI2, and $\bar{B(v)}$ is the mean background activity concentration.

## 3 RESULTS

### 3.1 Study of expansion size with no background activity

**FIGURE 2:**
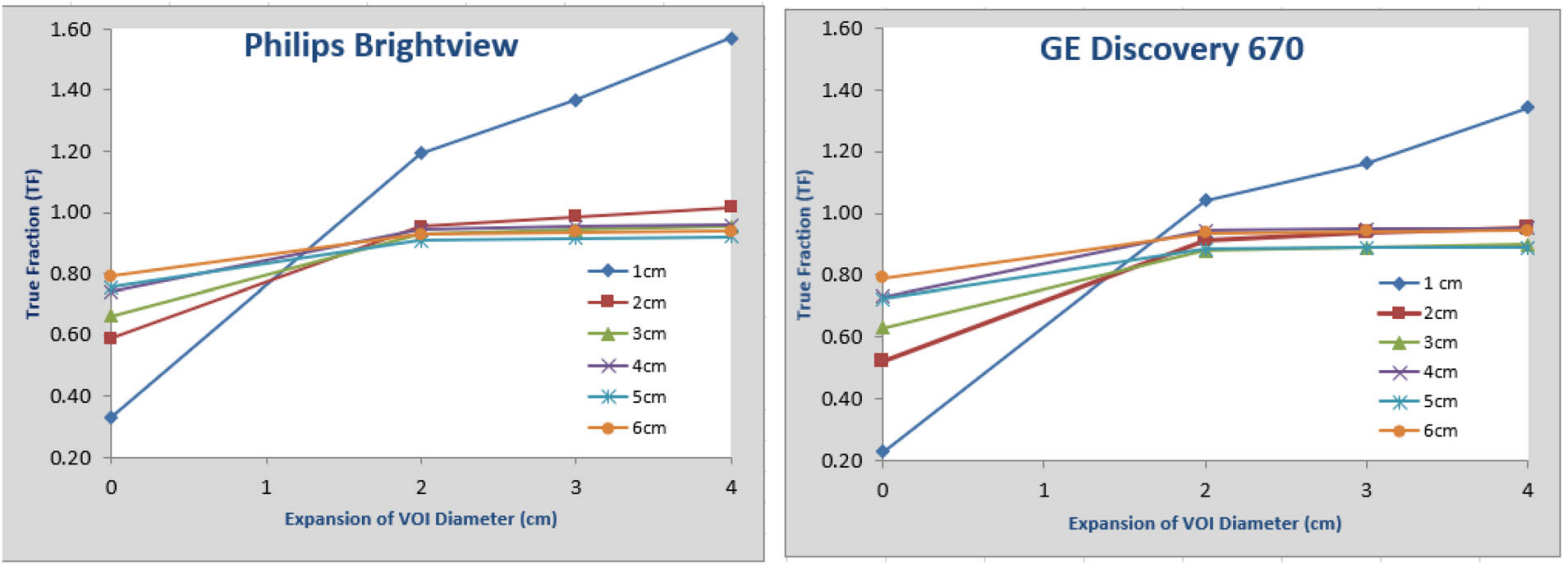
Study of the effect of expansion size on the total activity accuracy using data with no background activity for Philips Brightview (left) and GE Discovery (right): graphs above show true fractions (TF) versus expanded volume‐of‐interest (VOI) diameter for each sphere from 0 cm (matching sphere dimension) to 4 cm. The TF at 0 cm corresponds to the traditional method for measuring a recovery coefficient. Different lines correspond to spheres of different diameters from 1 to 6 cm.

We first performed a study to determine the expansion size of the VOI using data without any background. We studied the effect of expansion size on the total activity estimation accuracy by using different size of expansions. Figure 2 shows the results with expansions of diameters by 0–2–3–4 cm for spheres of diameters from 1 to 6 cm. The true fraction (TF) is defined by the measured total activity divided by the calculated (true) total activity. The TF at 0 cm corresponds to traditional method: recovery coefficient. It can be seen that traditional recovery coefficient not only depends on the size of the object but also varies slightly between the two systems. The spill‐out activities are still very significant even for large spheres/objects (as much as 20% for 6‐cm sphere) based on the results displayed. It can also be observed that an expansion of about 2 cm is able to capture almost all the activity, and expansions beyond 2 cm provide very small improvements for quantitative accuracy.

Based on this study, the expansion of diameter was set to be 2 cm for different spheres for our proposed methodology. The 2 cm expansion is possibly related to the spatial resolution of the SPECT system, which include the effects of the system hardware (crystal thickness, PMT, positioning algorithm, collimator, photon energy, etc.), acquisition protocol (detector radius of rotation and acquisition pixel size), and the reconstruction (pixel size, reconstruction algorithm, and smoothing filter). Both systems studied had very similar characteristics. It can be observed that the 1 cm sphere is an outlier among the six spheres. After further examination, we found that two factors contribute to this. The first factor is the limitation of 3D printing. The opaque stem and sphere made it difficult to observe the liquid level in the sphere, especially for the 1 cm sphere. This has resulted large portion of residual activity in the stem during filling of 1‐cm sphere. The second factor is the small volume of 1‐cm sphere. The small volume makes the activity in the stem a significant portion of the total activity. Because of the previous limitation of our 3D printing, meaningful conclusions can only be drawn from the other five spheres.

### 3.2 Study of total activity estimation with different background activities

**FIGURE 3:**
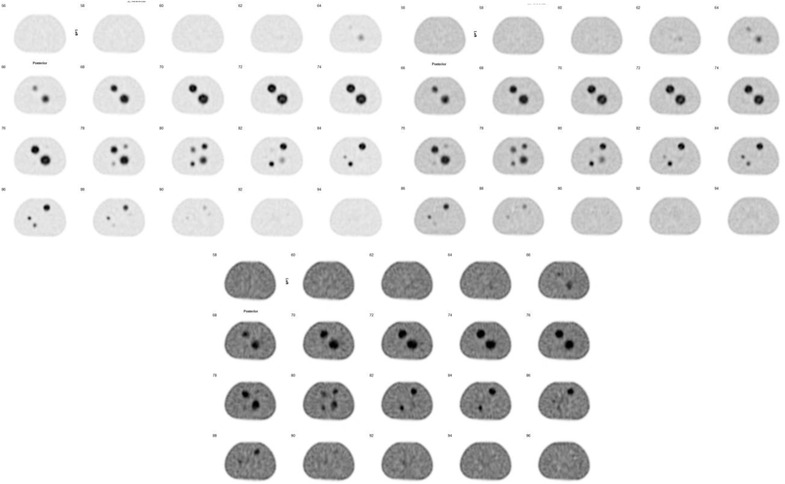
Reconstructed SPECT images with Hermes standardized uptake value (SUV)‐SPECT for data of sphere/background activity concentration ratio of 8:1 (top left), 4:1 (top right), 2:1 (bottom) acquired with Philips Brightview system

The proposed quantification method described in Section 2 is then used to analyze the 12 sets of reconstructed images. In Figure 3, we display images reconstructed with Hermes SUV‐SPECT with sphere/background activity ratios of 8:1, 4:1, and 2:1 from SPECT data acquired with Philips Brightview. The images reconstructed from GE data by GE Q.Metrix or Hermes SUV‐SPECT are similar to images reconstructed by Hermes SUV‐SPECT. It can be observed that significant artifacts (streaks and non‐uniformity) are present in the reconstruction images from sphere/background activity ratio of 2:1 for both systems. This could be attributed to inadequate detector uniformity at high count rates and scatter correction and count loss due to detector dead time because the count rate of the 2:1 data acquisition is much larger than a regular clinical scan, and the vendor's scatter and uniformity correction algorithm may not be able to correct the scatter/nonuniformity adequately, which degraded the image quality. However, this does not mean quantitative SPECT will not work for sphere/background activity ratio of 2:1 even if the count rate is within the range of clinical scan.

**FIGURE 4:**
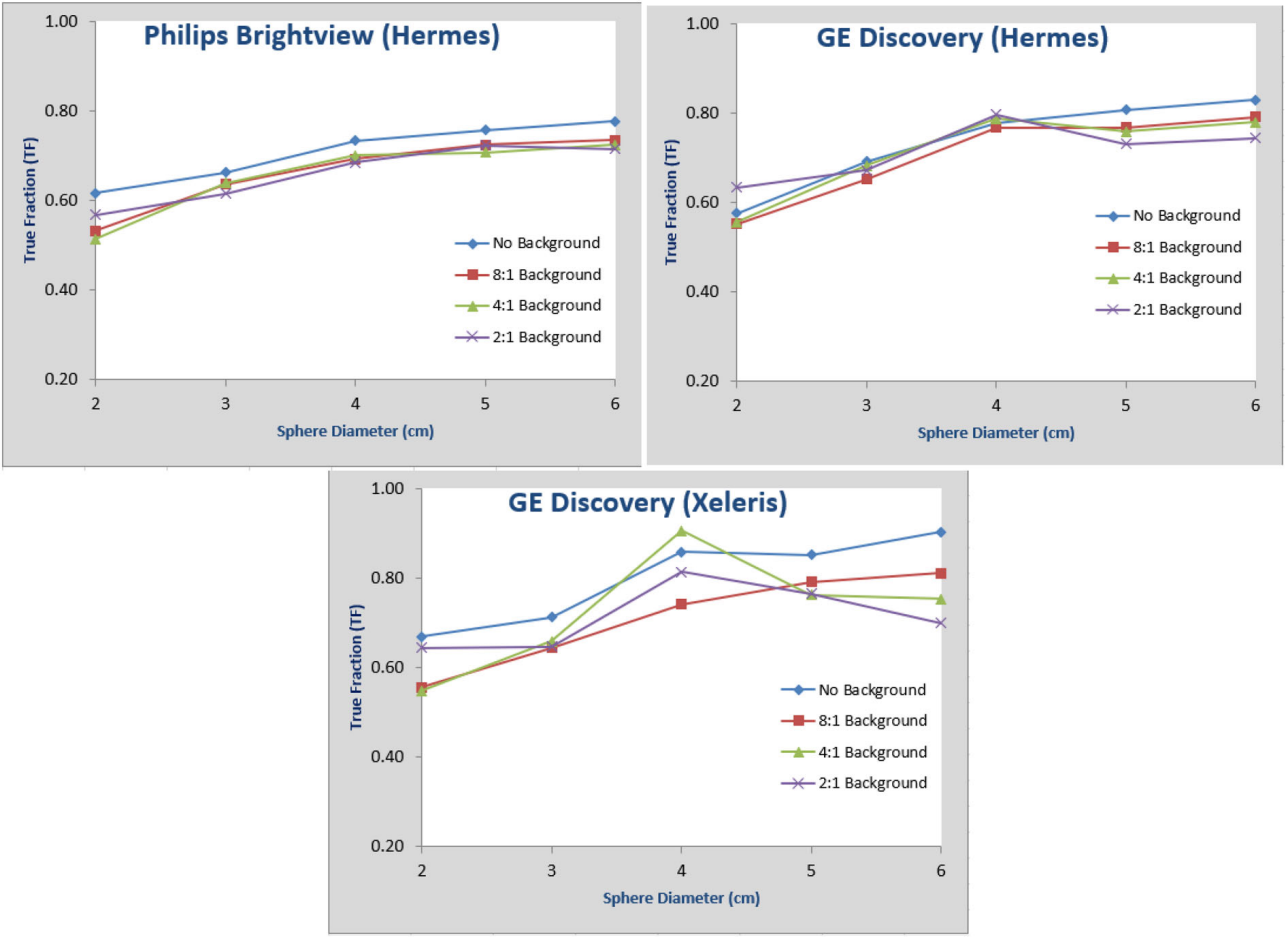
Plots of recovery coefficients (true fraction without volume expansion) versus sphere sizes for Philips‐Hermes, GE‐Hermes, and GE‐Xeleris for SPECT images reconstructed from data sets of sphere/background activity concentration ratio of 8:1, 4:1, 2:1, and no background activity

In Figure 4, we plotted the recovery coefficients (which are the TF without volume expansion) versus sphere sizes for different data sets. It can be observed that the results are size‐dependent and vary slightly between the two systems, which is similar to the results described in Ref. [13].

**FIGURE 5:**
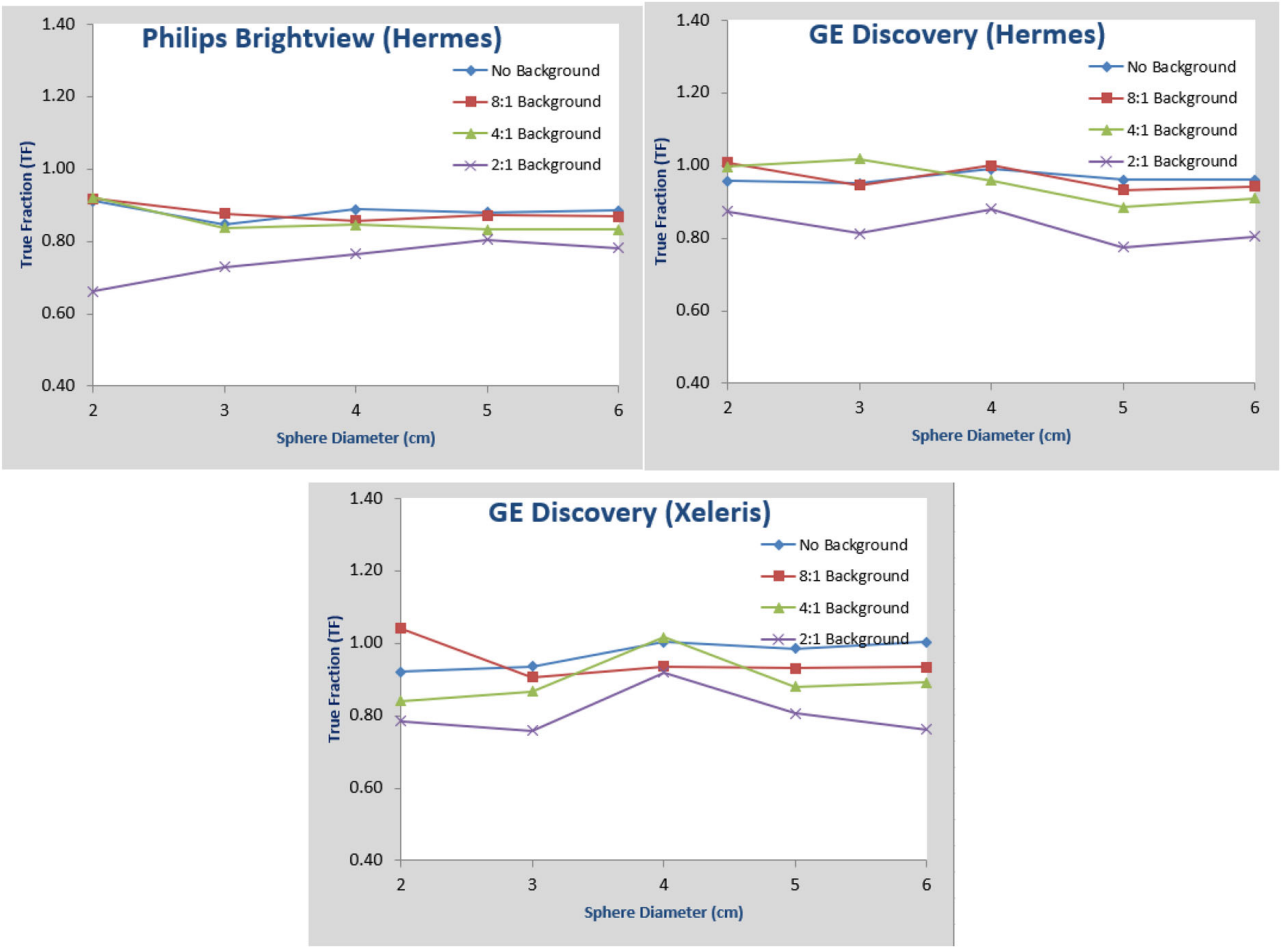
Plots of true fraction (TF) versus sphere sizes for Philips‐Hermes, GE‐Hermes, and GE‐Xeleris for SPECT images reconstructed from data sets of sphere/background activity concentration ratio of 8:1, 4:1, 2:1, and no background activity

**FIGURE 6:**
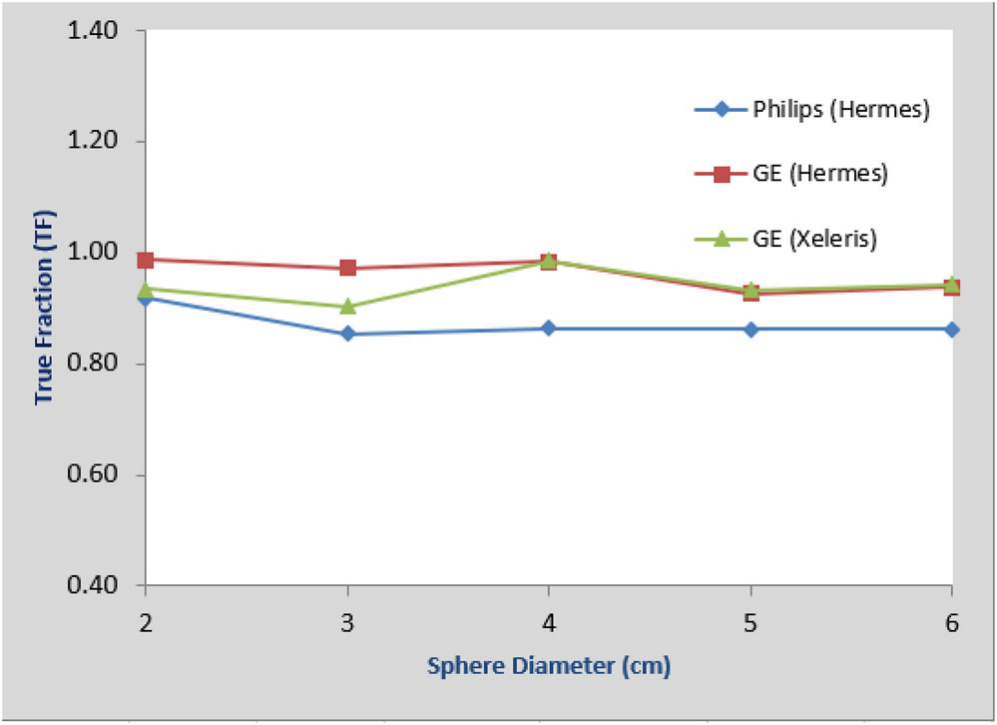
Plots of true fraction (TF) averaged over data sets of spheres to background activity concentration ratio of 8:1, 4:1, and no background versus sphere sizes from SPECT reconstruction images for Philips‐Hermes, GE‐Hermes, and Ge‐Xeleris

In Figure 5, we plotted the calculated TF with 2 cm volume expansion and with background correction applied as defined earlier versus sphere sizes for different data sets. It can be observed that the activity estimation accuracy from sphere/background ratio of 2:1 is worse than that from data sets of 8:1 and 4:1 ratios, and no background activity. As discussed previously, this is mainly caused by the unrealistically high background, which degraded the image quality. For summary, we also plotted in Figure 6 the average TF for data sets of 8:1, 4:1, and no background for the three different combinations. It can be observed both here and from the original plots in Figure 5 that for sphere sizes of 2–3–4–5–6 cm, the total activity estimation accuracy is relatively stable across different sphere sizes and analysis combinations.

## 4 DISCUSSION

This manuscript demonstrates a robust method to estimate the total activity for objects of different sizes across different quantitative SPECT reconstructions. The advantage of the proposed method over sphere‐based recovery coefficients is that the latter heavily depends on the size of the objects and also varies slightly from system to system. The proposed method is consistent across objects of different sizes and for the two clinical systems that were studied. From the total activity, the average activity can be estimated by dividing the total activity with the volume. However, the quantitative accuracy of activity distribution (or SUV) within the volume still needs further validation.

**FIGURE 7:**
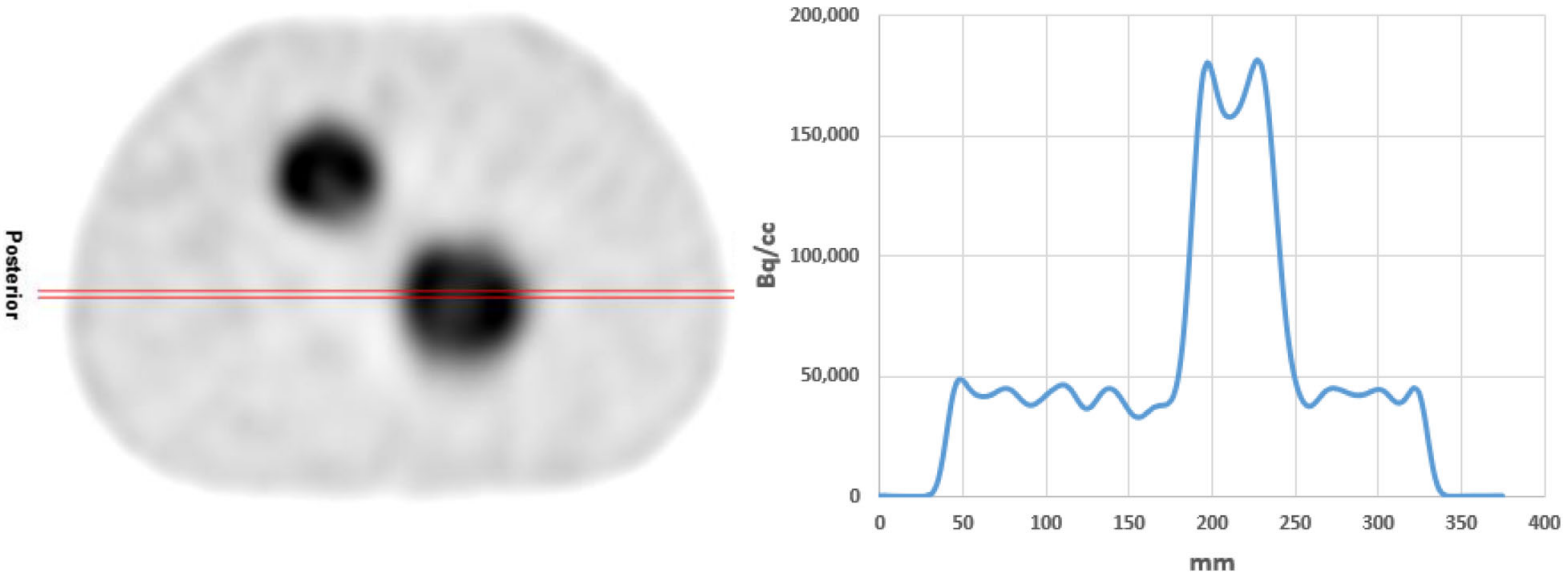
Edge enhancement effects shown in the reconstructed SPECT images resulted from resolution recovery: on the left is the SPECT reconstruction from GE Hermes reconstruction, on the right is the profile plotted along the red line of the SPECT image. Edge enhancement can be observed near the boundary of the sphere. It is worth noting that white means smaller counts in the image.

Although resolution recovery improved the overall image quality of the SPECT image, significant edge enhancement effects can be observed in the reconstruction images. In Figure 7, we displayed the sphere SPECT reconstruction images along with the profile across one of the spheres; it can be clearly observed that the edge of the sphere in the reconstruction has much higher activity concentration than other parts of the sphere, although the concentration should be uniform. As the results in this manuscript show, the resulted nonuniformity from edge enhancement did not affect the total activity estimation accuracy, but it will affect the accuracy of pixel‐based quantitative SPECT.

The accuracy of the proposed method will depend on how well the VOI can be delineated. The spherical objects in our experiment have relatively simple shapes, and we are able to accurately position the VOIs, and we know the activity is confined in the spheres. Expansion of the VOI's is simply accomplished by increasing the VOI diameter. For irregular‐shaped objects, the delineation will be more difficult. With the available CT images, we can always use the CT images as a reference in the segmentation process as long as the object boundary is visualized. Accurate expansion of an irregularly shaped VOI may be more difficult. The existing visualization tools incorporate an expansion VOI tool but only by increasing the VOI size by adding one voxel at a time at its periphery. This may be reasonably accurate for large objects, but not for small objects. This was the case in our experiment using the GE Xeleris by which we were only able to expand VOI's using the voxel approach. The results in Figures 4 and 5 show a larger variation in the GE Xeleris graph as compared to Hermes measurements. The Hermes tool allowed us to redraw a VOI by specifying its diameter. It is expected that an expansion algorithm that incorporates a measure of object boundary curvature would yield more accurate results. With proper placement of a VOI of an irregularly shaped object at its boundary and a robust tool to expand that VOI, the quantification of activity is postulated to be as accurate as for spheres.

Sometimes two lesions may be close to each other, and there may not be enough space for expansion between the lesions. This situation will need further study. Either the expansion size needs to be reduced or the two lesions can be included in one VOI.

The expansion method requires a background correction applied to the expanded volume used to capture all of the spill‐out activity. For our experiment, it was easy to enlarge the sphere an additional cm to measure the background concentration. It is not expected that the same method would be always appropriate for patient images. What is required is a proper sampling of the patient background in the vicinity of the object or tumor. A VOI off to the side may be sufficient. Likewise in quantifying activity in planar imaging studies, a background is drawn near or adjacent to the ROI surrounding the object of interest. The most accurate background sampling is by drawing a background ROI almost encircling the drawn ROI. The same principle can be applied in 3D or tomographic image quantification.

The main purpose of this manuscript is to develop a method to quantify the total activity in a quantitative SPECT reconstruction and validate the activity estimation accuracy with known truth values. That is why we focused on phantom studies in this manuscript. The same method can be extended to patient studies as well with the addition of segmentation tools.

It seems even though the CT image quality of the Brightview system is not as good as a diagnostic CT system such as GE Discovery, that did not affect the overall total activity estimation accuracy. This is mainly because we used CT images for attenuation correction and segmentation reference. The CT image quality of the Brightview system seems to be sufficient for these two purposes.

## 5 CONCLUSIONS

The results demonstrate that the proposed quantitative method is robust for objects of different sizes. This method has the potential to be robust in performing quantitative analysis of organ and lesion activity in patients for clinical SPECT/CT images using the expanded VOI technique. The results also demonstrated that the current quantitative SPECT packages (Hermes SUV‐SPECT and GE Q.Metrix) are reasonably accurate when used for estimating the total activity of VOIs with the proposed technique.

## AUTHOR CONTRIBUTIONS

Junguo Bian, experiment design, data acquisition and analysis, conceptualization, results analysis, writing‐original draft, writing‐revision and response; Judy James, data analysis, conceptualization, editing the original draft; Robert Wagner, phantom design, editing original draft and revision; James Halama, phantom design, experiment design, data acquisition and analysis, conceptualization, data analysis, critical editing the original draft and revision.

## CONFLICT OF INTEREST

The authors have no potential conflict of interest to disclose.
